# Centrality dependency of proton, deuteron, and triton’s temperatures in Au+Au collisions at 200 GeV

**DOI:** 10.1038/s41598-024-55759-2

**Published:** 2024-05-04

**Authors:** Imran Khan, Abdul Qudus, Moustafa Salouci, Abd Haj Ismail

**Affiliations:** 1 Department of Physics, University of Science and Technology, Bannu, KP 28100 Pakistan; 2 Government Degree College, Serai Naurang, Lakki Marwat, KP Pakistan; 3 Strategic Planning and Institutional Identity Administration, King Faisal University, Al-Ahsaa, 31982 Kingdom of Saudi Arabia; 4 College of Humanities and Sciences, Ajman University, PO Box 346, Ajman, UAE

**Keywords:** Nuclear physics, Particle physics

## Abstract

The transverse momentum (*p*_T_) spectra of protons (p), deuterons (d), and tritons (t) in 200 GeV gold–gold (Au + Au) collisions at RHIC are examined across a range of centrality bins using the Levy Tsallis (TS) statistical model. The model's predictions closely match the experimental results from PHENIX (p) and STAR (d and t) Collaborations. Kinetic freeze-out temperatures of hadrons are obtained from particle spectra. The results showed that the kinetic freeze-out temperature decreases as collisions move from center to the periphery. This work found mass-dependent kinetic freeze-out temperatures, heavier particles arrive to the freeze-out phase before lighter ones. Comparison with same data fitted by blast wave function with Tsallis statistics (BWTS) showed that *T*_0_ values are increasing from central to peripheral collisions, while in case of TS function (current analysis) it decreases. This behavior puts a question mark on the reliability of using such functions for temperature extraction.

## Introduction

Chemical and kinetic freeze-out temperatures are two critical stages that the system goes through. The various levels of system excitation are reflected in these stages, represented by chemical and kinetic freeze-out temperatures. Usually, during chemical freeze-out, ratios of particle multiplicities get fixed. Using the thermal model, the chemical freeze-out temperature is calculated from different particle ratios^1–3^. The transverse momentum spectra of many particles are determined at the stage of kinetic freeze-out. These spectra can be used to calculate the thermal or kinetic freeze-out temperature (*T*_0_) using the hydrodynamical model^4^.

Thermal motion and transverse flow velocity are included in the low-*p*_T_ region of transverse momentum spectra. In contrast to thermal motion, which represents excitation, transverse flow velocity denotes system expansion. Random thermal motion is isolated to ascertain the kinetic freeze-out temperature (*T*_o_) by removing the influence of transverse flow velocity (*β*_T_). To address this, techniques such as Boltzmann Gibbs statistics in blast wave fit^5–7^, and blast wave model using Tsallis statistics (BWTS)^8–10^ are often used. Similarly Levy Tsallis fitting function (TS)^11^ and other functions, including those presented in^12–18^ are also used to get the temperature of hadrons by studying their transverse momentum (*p*_T_) or transverse mass (*m*_T_) spectra.

The situation is difficult because of the pivotal relationship between *T*_o_ and centrality. From central to peripheral collisions, *T*_o_ reduces^19–22^ and increases^23–25^, respectively. Each perspective provides a justification. A lower *T*_o_ in center collisions denotes a longer-lasting fireball in such cases, but a higher *T*_o_ in core collisions denotes a more intense system excitation as a result of more energetic collisions. Which collision system exhibits the greater *T*_o_ must be determined. Particle freeze-out has been viewed from a variety of angles, including those that cover single, double, and multiple kinetic freeze-out scenarios. The particular freeze-out situation must be identified.

The main objective of this investigation is to derive temperatures from the *p*_T_ spectra of protons, deuterons, and tritons, to check the behavior of the *T*_o_ with centrality and compare the results with those obtained in reference^25^. In both cases same experimental data has been used for analysis, that is, protons, deuterons, and tritons obtained in Au+Au collisions at 200 GeV at the same conditions. The experimental data is obtained from published works^26–28^. In this case we have used Levy Tsallis model fitting function (Eq. 1) for extracting freeze-out temperatures of said hadrons. Then, the results have been compared with reference^25^ results in which blast wave model using Tsallis statistics (Eq. 2) is used under the same conditions. We have obtained some striking results.

We know that deuterons and tritons are light nuclei, so it is difficult to understand how they arise in relativistic heavy ion collisions^29–31^. Nucleon and anti-nucleon coalescence is one potential scenario^32–36^. Light nuclei cannot withstand temperatures much higher than their binding energies (1.1 MeV/nucleon for d and 2.93 MeV/nucleon for t). Given that light hadrons typically have kinetic freeze-out temperatures of 100 MeV^37^, it is conceivable that once nucleons are released from the hot, dense system, they will dissociate before recombining to create new particles. Therefore, light nuclei analysis can provide more information on the nucleon distribution at freeze-out^32,35,38^.

The importance of other quantities such as, volume in high-energy collisions must be emphasized before diving into the formalism. Freeze-out state is defined as the moment when elastic scattering between particles ceases and only coulombic force remains interactive. The region occupied by the particles at this time is known as the kinetic freeze-out volume (*V*). Different freeze-out volumes could appear at different periods. The derivation of multiplicity, micro-canonical heat capacity, and the negative branch or shape of caloric curves within thermal conditions all depend critically on this quantity, which provides insights into the existence of phase transitions.

"The method and formalism" deals with the method and mathematical formalism, whereas "Results and discussion" presents the results and the discussion that follows. “Conclusion” provides an overview of our key conclusions and suggested actions.

## The method and formalism

Particles are created in high-energy collisions through both soft and hard processes. These processes can be modeled using a variety of methods, including the standard distribution^39,40^, the blast wave model using Boltzmann Gibbs statistics^5–7^, and the Hagedorn thermal model^21^. The list also contains Levy Tsallis function^11^ and blast wave model using Tsallis statistics^25^. Effective temperature (*T*_eff_), initial temperature (*T*_i_), thermal or kinetic freeze-out temperature (*T*_0_), thermal freeze-out volume (*V*) of the interacting system, and transverse flow velocity (*β*_T_) of the final-state particles are just a few of the parameters that can be understood by analyzing the *p*_T_ spectra of the particles^11^. To extract these details, we use a fitting strategy that makes use of several models and distributions. For this reason, we use Tsallis statistics in the current study.

Use of the Levy Tsallis function^11^ and its results comparison to blast wave model using Tsallis statistics is the main topic of this work. Levy Tsallis function is given by;

1 $$ f(p_{T} ) = p_{T} \times \frac{N(n - 1)(n - 2)}{{nT_{0} \;\left[ {nT_{0} + m_{0} (n - 2)} \right]}} \times \left( {1 + \frac{{m_{T} - m_{0} }}{{nT_{0} }}} \right)^{ - n} $$

Here *f* $\left({p}_{T}\right)=\frac{1}{2\pi N}\frac{{d}^{2}N}{d{p}_{T}dy}$ , *T*_0_ is the freeze-out temperature of the hadrons, *N* is the normalization constant, *n* is the entropy index and *m*_T_ and *m*_0_ are the transverse mass and the rest mass of the hadron, respectively. It works both at low and high *p*_T_ regions of the *p*_T_ spectra of hadrons.

Both Levy Tsallis function and Blast wave model using Tsallis statistics have been extensively used to get the kinetic freeze-out temperatures of hadrons for decades^8,11,25^. It is used in^25^ in which Au+Au collisions are studied for the production of protons, deuterons, and tritons. According to^8^, the blast wave fit using Tsallis statistics is used to derive the probability density function.

$$f\left({p}_{T}\right)\propto {m}_{T}{\int }_{-Y}^{+Y}{\text{cosh}}(y)dy{\int }_{-\pi }^{\pi }d\Phi {\int }_{0}^{R}rdr{\left[1+\frac{(q-1)\left\{{m}_{T}{\text{cosh}}(y)cosh\left(\rho \right)-{p}_{T}sinh\left(\rho \right)cos\left(\Phi \right)\right\}}{{T}_{o}}\right]}^{-1/(q-1)}$$

This gives

2 $$f\left({p}_{T}\right)=C\frac{gV}{{\left(2\pi \right)}^{2}}{p}_{T}{m}_{T}{\int }_{-Y}^{+Y}{\text{cosh}}(y)dy{\int }_{-\pi }^{\pi }d\Phi {\int }_{0}^{R}rdr{\left[1+\frac{(q-1)\left\{{m}_{T}{\text{cosh}}(y)cosh\left(\rho \right)-{p}_{T}sinh\left(\rho \right)cos\left(\Phi \right)\right\}}{{T}_{o}}\right]}^{-1/(q-1)}$$

where *C* is normalization constant, which leads the integral in Eq. (2), to be normalized to 1, *g* is the degeneracy factor which is different for different particles based on *g*_*n*_ = 2*S*_*n*_ + 1, *m*_*T*_ is the transverse mass, that is *m*_*T*_ = $\sqrt{{p}_{T}^{2}+{m}_{o}^{2}}$. Here *m*_0_ is rest mass of the particle, *Φ is* the azimuthal angle, *r* is the radial coordinate, *R* is the maximum *r*, *q* is the measure of degree of deviation of the system from an equilibrium state, *ρ* = tanh^−1^{*β*(*r*)} is the boost angle. Here {*β*(*r*)} = *βS*(r/*R*)^no^ is a self-similar flow profile in which *β*_S_ is the flow velocity on the surface, as a mean of *β*(r), *β*(*r*) = (2/*R*^2^) ${\int }_{0}^{r}r\beta \left(r\right)dr$= 2*βs*/(*n*_0_ + 2) = 2*βs*/3, and *n*_0_ = 1, furthermore, the index −1/(*q* – 1) in Eq. (1) can be replaced by −*q*/(*q* – 1), because *q* is close to 1. This replacing results in a small and negligible divergence in the Tsallis distribution.

References^11,16,19–22,25,37–43^ contain further information regarding the fitting parameters and procedure.

### Ethical approval

The authors affirm that they have adhered to all ethical standards with regard to the topic of this study.

## Results and discussion

**Figure 1:**
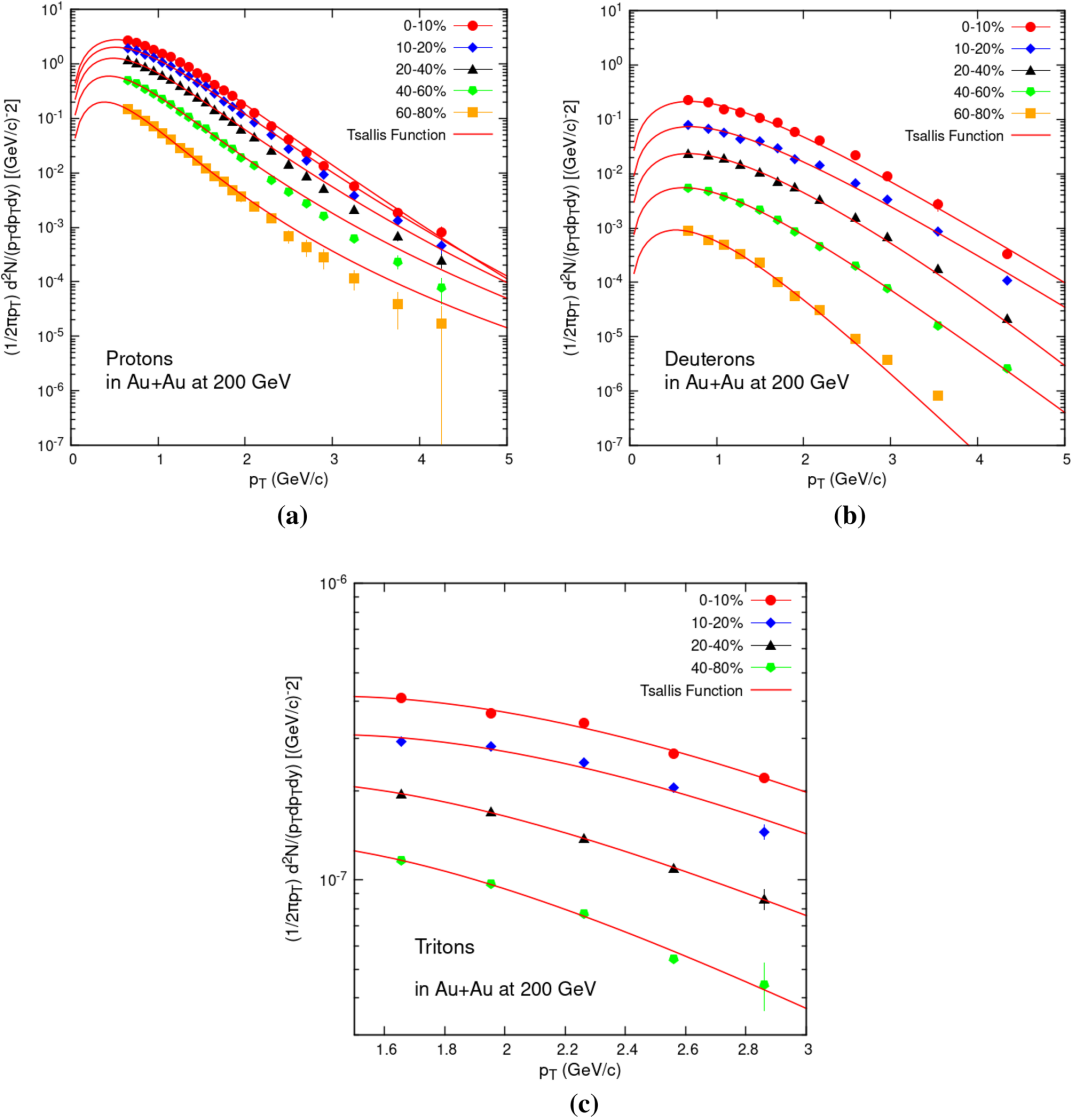
Transverse momentum spectra of protons (**a**), deuterons (**b**), and tritons (**c**) produced in Au+Au collisions at $\sqrt{{s}_{NN}}$ = 200 GeV. Solid curves are fitting results from Levy Tsallis function Eq. (1).

The transverse momentum (*p*_T_) distributions for protons, deuterons, and tritons in Au+Au collisions at $\sqrt{{s}_{NN}}$ = 200 GeV are shown in Fig. 1 in the representation {($\frac{1}{2\pi {p}_{T}}$) $\frac{{d}^{2}N}{dyd{p}_{T}}$}. The data were taken from^26^ (protons),^27^ (deuterons), and^28^ (tritons). The centrality intervals for the *p*_T_ distributions of protons and deuterons are 0–10%, 10–20%, 20–40%, 40–60%, and 60–80%. Triton centrality bins are from 0 to 10%, 10 to 20%, 20 to 40% and 40 to 80%, all spectra are at mid-rapidity (y). The symbols show the experimental data, and the curves represent our fitting using the Levy Tsallis function (Eq. 1). Table 1 shows the extracted fit parameters and their uncertainties. It is clear from Fig. 1a–c that Eq. (1) accurately passes through the transverse momentum spectra of all the three hadrons obtained in Au+Au collisions at $\sqrt{{s}_{NN}}$ = 200 GeV.

**Table 1:** Values of variables in Eq. (1) obtained from Fig. 1.

Particle	Centrality (%)	*N* (GeV)	*N* error	*T* (GeV)	*T* error	*n*	*n* error	*Chi* square/n.d.f
Proton	0–10	0.942686	0.01971	0.25335	0.0062	27.927	0.355	5.01E−04
	10–20	0.483303	0.01074	0.23718	0.0064	19.511	0.561	2.52E−04
	20–40	0.320094	0.0074	0.21431	0.0055	14.703	0.472	8.14E−05
	40–60	0.245373	0.0049	0.17496	0.0036	10.424	0.881	6.85E−06
	60–80	0.092968	0.00245	0.13669	0.0037	8.128	0.557	5.35E−07
Deuteron	0–10	0.810737	0.0523	0.40592	0.0016	64.185	0.201	7.94E−05
	10–20	0.789703	0.01716	0.40749	0.0055	58.071	0.081	8.98E−06
	20–40	0.743625	0.0405	0.38066	0.0089	50.498	0.892	1.26E−07
	40–60	0.716768	0.08606	0.34131	0.0085	46.365	0.058	4.53E−09
	60–80	0.68675	0.06406	0.26376	0.0076	35.632	0.071	1.05E−09
Triton	0–10	0.5307	0.09308	0.64285	0.0913	76.712	0.831	2.45E−06
	10–20	0.4706	0.06438	0.63363	0.0531	64.161	0.311	1.44E−06
	20–40	0.4389	0.03209	0.45529	0.0245	53.059	0.498	8.31E−09
	40–80	0.3998	0.01228	0.40359	0.0106	48.376	0.542	7.74E−08

**Figure 2:**
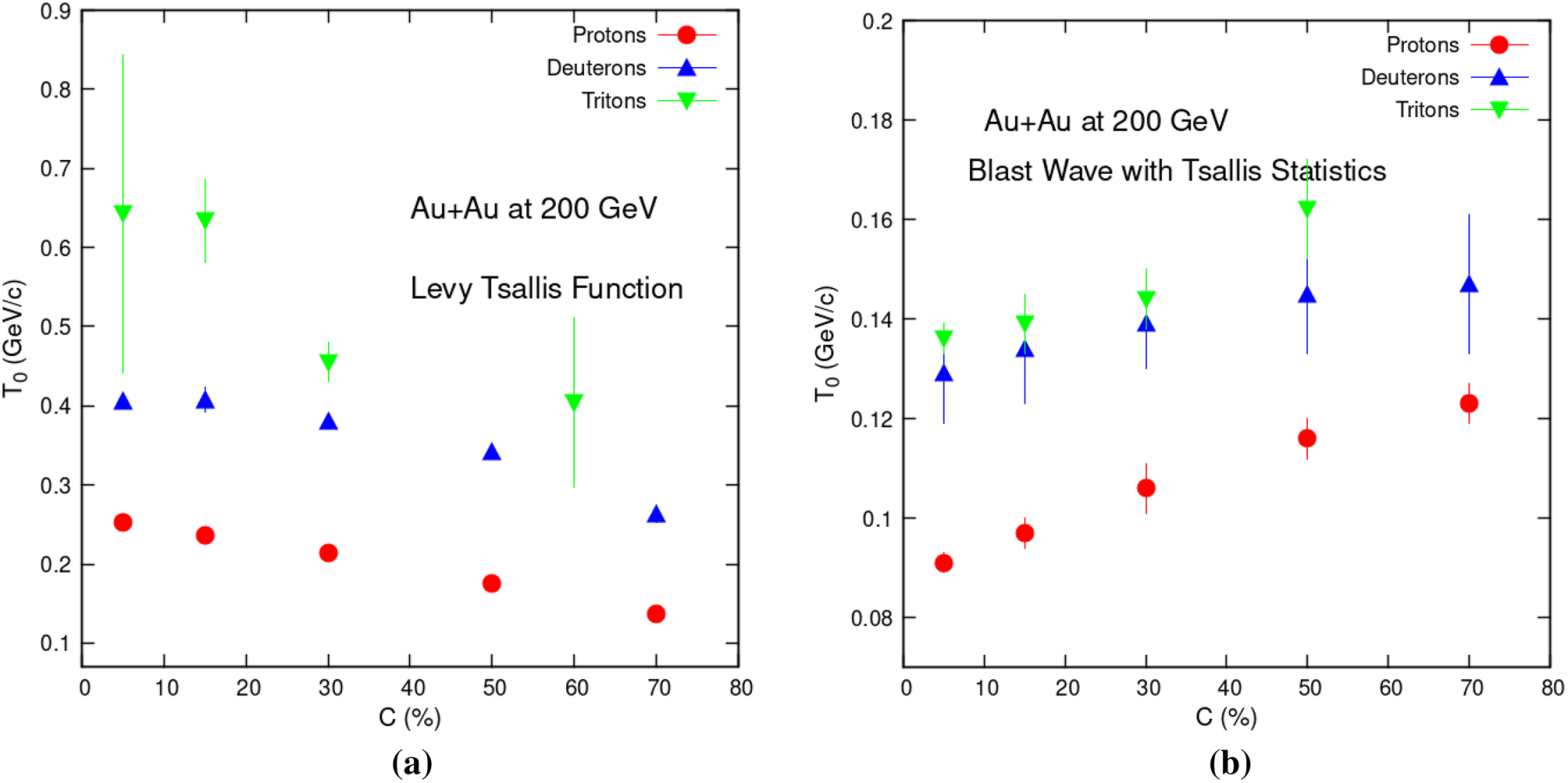
Centrality dependency of *T*_0_ obtained via Levy Tsallis function (left) current analysis (Eq. 1), and blast wave function with Tsallis statistics (right) (Eq. 2)^25^.

To highlight parameters trends, Fig. 2 shows how the kinetic freeze-out temperature (*T*_0_) varies with respect to centrality. Figure 2a contains results from Eq. (1) which is current study, while Fig. 2b has results from Eq. (2) studied in reference^25^. It is noteworthy that, in current analysis, *T*_0_ is significantly higher in central collisions and gradually decreases with diminishing centrality. This denotes a change from center to periphery collisions and a longer lifetime for the fireball. However in case of Fig. 2b we see opposite trends. *T*_0_ seems to be significantly lower in central collisions and steadily increases with lessening centrality. Such behavior is also reported in following references: decreasing^19–22^ and increasing^23–25^. This trend puts a big question mark on the credibility of use of such functions for temperature extraction. However we can expect such behavior if we consider the physical meaning of both fit functions. We have used Tsallis statistics, so both include possible deviation of the data from the equilibrium Boltzmann distribution. But only BWTS takes into account the radial flow of the particles. Nothing is referring to the radial flow in the TS formula. Since we know that the transverse momentum distribution's slope is affected by the temperature and the radial flow. Radial flow pushes the particles towards higher transverse momentum, making the spectrum less steep. So, if the radial flow is present in experimental data, but the fitted function does not include the radial flow, then the extracted slope parameter will be affected. If the intuition from the Boltzmann distribution can be transferred to the Tsallis distribution, then the parameter *T*_0_ gets artificially larger because the radial flow is not considered. And this is what we indeed see in this research article.

Also it can be observed that *T*_0_ has a mass-dependent pattern as well, with tritons having the highest value, followed by deuterons, then protons. This means that heavier particles arrive to the freeze-out phase before their lighter counter parts do. *T*_0_ versus mass-dependency has same behavior in both cases.

Table 1 also reveals that *T*_0_ values obtained via Eq. (1) are greater than those obtained via Eq. (2). Greatest value for triton emission in central collisions is 642 ± 91 MeV in case of TS fittings. It is very much greater than 160 ± 20 MeV obtained in case of BWTS equation for most peripheral collisions.

**Figure 3:**
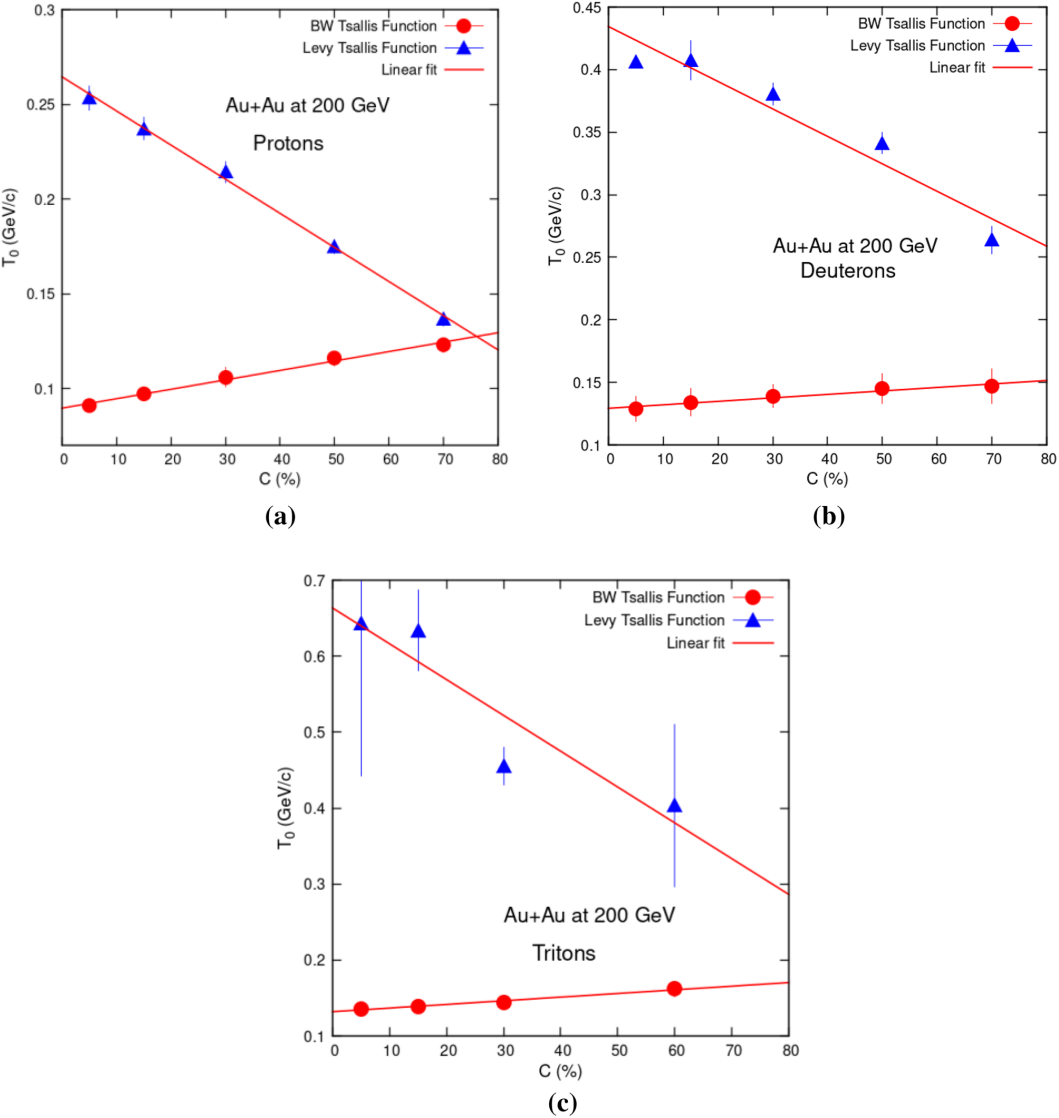
Comparison of centrality dependency of *T*_0_ for protons (**a**), deuterons (**b**) and tritons (**c**) obtained via fittings of Levy Tsallis function (Eq. 1) (present work) and blast wave function with Tsallis statistics (Eq. 2)^25^. Red solid line is the linear function (*T*_0_ = *aC* + *b*) fit curve.

Figure 3 shows the same information in a different way. Data from both Tsallis fitting models have been put on single place for each hadron. Figure 3 shows a trend of *T*_0_ = *aC* + *b*, with *C—*centrality, *a*, *b—*free parameters. Here *a* is the slope of the curve, which shows how fast or slow variation in *T*_0_ takes place, as we go from central to peripheral collisions. Values of variables *a* and *b* are given in Table 2. Slopes of hadrons in the case of TS fittings are negative while positive in the case of BWTS function. TS slopes are much steep as compared to those of BWTS function. Decrease in *T*_0_ values for hadrons is greater in TS case than increase in values of *T*_0_ in BWTS case. Decrease in slopes of hadrons is also particle mass dependent. Greater the mass, greater is the reduction slope.

**Table 2:** Values of variables *a* and *b* extracted from linear fit equation, *T*_0_ = *aC* + *b*.

Function	Particle	*a*	*a* error	*B*	*b* error	Reduced Chi square
Levy Tsallis (Eq. 1)	Protons	−0.00180172	5.23e−05	0.264562	0.002163	7.5762e−06
	Deuterons	−0.00219504	0.0003564	0.434466	0.01474	0.000351802
	Tritons	−0.00471279	0.00139	0.663449	0.04789	0.00333151
Blast wave Tsallis (Eq. 2)	Protons	0.00049742	3.041e−05	0.089685	0.001257	2.56077e−06
	Deuterons	0.00027761	3.69e−05	0.129361	0.001526	3.77076e−06
	Tritons	0.00047971	5.327e−05	0.132058	0.001836	4.89493e−06

The values of *T*_0_ in the TS approach are, in most cases, higher (sometimes much) than the typical temperatures of chemical freeze-out obtained in the Statistical Hadronization Model approaches and the phase transition temperatures from the Quark-Gluon Plasma to the hadronic matter, calculated with Lattice QCD. This is hard to be expected. On the other hand, the temperatures of the kinetic freeze-out in the BWTS approach are in the same range or lower than the chemical freeze-out and the phase transition temperatures, which is much more intuitive.

**Figure 4:**
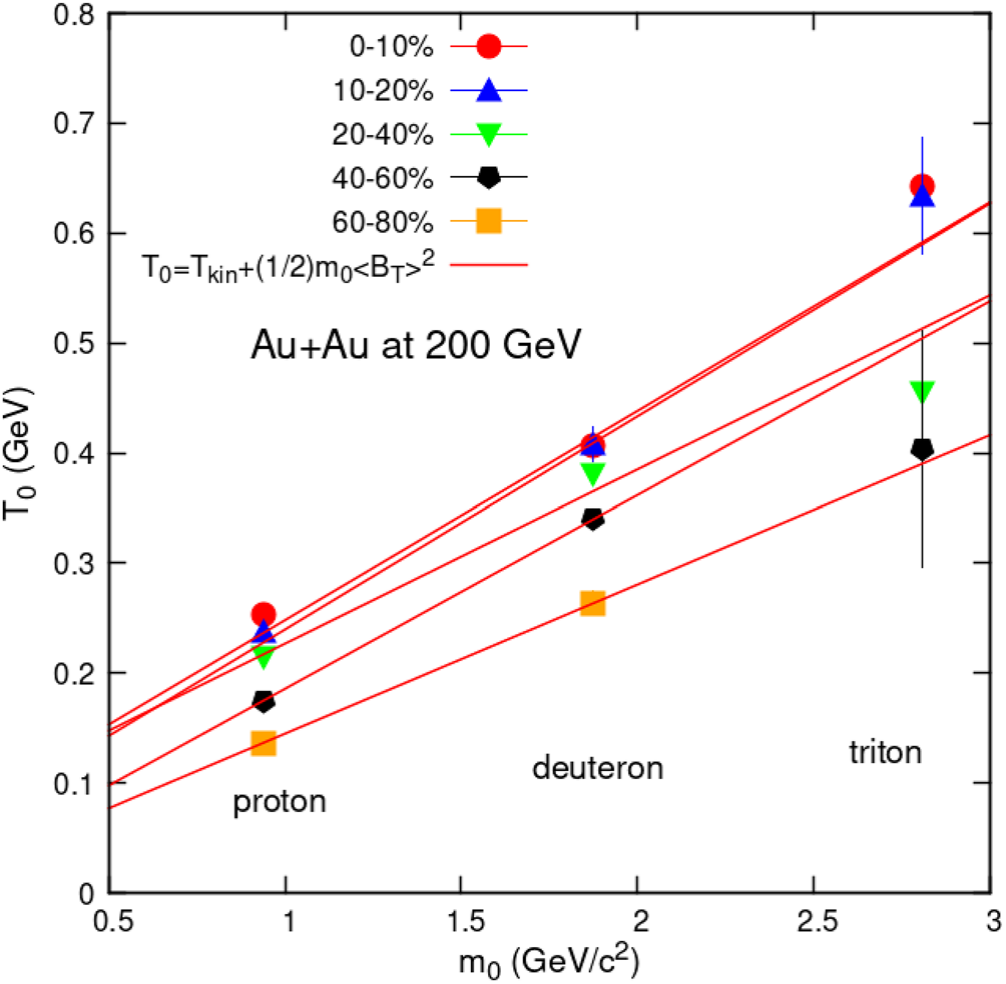
*T*_o_ Dependency on masses of the hadrons.

In fact, one can investigate this, using Fig. 4, which demonstrates that when particle mass increases, the parameter *T*_0_ rises. The temperature, *T*_0_, is essentially noticeably greater in central collisions and decreases toward the periphery.

With the Boltzmann statistics, when radial flow is not included in fits to transverse momentum spectra, there is a relation (Eq. 3) between the slope of the spectrum *T*_0_ , average transverse flow velocity $\langle \beta_{{\text{T}}} \rangle$  and the kinetic freeze-out temperature *T*_kin_:

3 $$ T_{0} = T_{kin} + \frac{1}{2}m_{0} \langle \beta_{{\text{T}}} \rangle^{2} $$

The formula is rough already with the Boltzmann statistics, so transferring it to the Tsallis one perhaps does not cause big additional error. Therefore, it is worth applying it to the data shown in Fig. 4 and comparing the results to the ones from BWTS^25^ in a slightly fairer way. Fitting of Eq. (3) gives the values of free parameters listed in Table 3.

**Table 3:** Values of variable $\langle \beta_{{\text{T}}} \rangle$ obtained from fit Eq. (3) and average *β*_T_ obtained using BWTS^25^.

Fit equation	Centrality (%)	$\langle \beta_{{\text{T}}} \rangle$ (c)	$\langle \beta_{{\text{T}}} \rangle$ error
Average *β*_T_ obtained using TS and Eq. (3)	0–10	0.622	0.006
	10–20	0.616	0.002
	20–40	0.562	0.007
	40–60	0.553	0.011
	60–80	0.521	0.009
Average *β*_T_ obtained using BWTS^25^	0–10	0.447	0.004
	10–20	0.421	0.012
	20–40	0.388	0.009
	40–70	0.355	0.007
	70–80	0.337	0.008

Comparison of values of variable, average transverse flow velocity of hadrons,  $\langle \beta_{{\text{T}}} \rangle$ obtained from fit Eq. (3) and average *β*_T_ obtained using BWTS^25^ given in Table 3, reveals that $\langle \beta_{{\text{T}}} \rangle$ decreases with centrality in both cases. However values of $\langle \beta_{{\text{T}}} \rangle$ obtained using Eq. (3) and TS equation are much higher as compared to those obtained via BWTS equation. It is important that their dependency on centrality is now of the same type i.e. both are decreasing. So we can say that both fitting functions, TS and BWTS may be used to extract temperature of hadrons.

**Figure 5:**
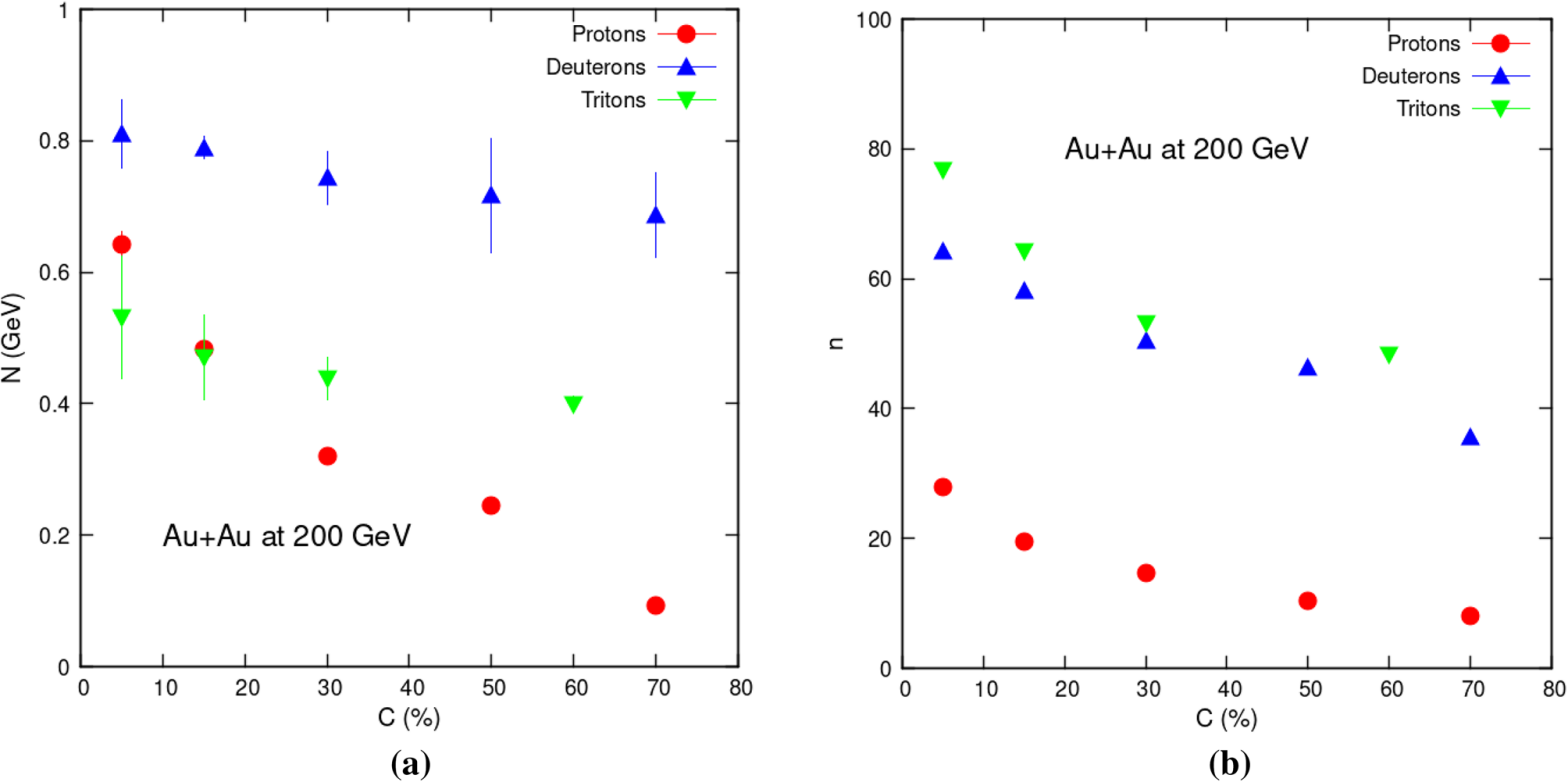
Variables “*N*” and “*n*” dependency on centrality.

Figure 5a shows relationship between *N* and centrality, whereas Fig. 5b shows the relationship between *n* and centrality. It is obvious that a drop in centrality causes a drop in *N* and *n* values. This can be explained by the system's internal interactions, which become more intense in central collisions. This effect causes a spike in central collision part.

## Conclusion

In order to extract the kinetic freeze-out temperature the study of transverse momentum spectra of protons (p), deuterons (d), and tritons (t) has been done via using the Levy Tsallis statistics. Levy Tsallis equation accurately passed through the *p*_T_ spectra of all three hadrons obtained in Au+Au collisions at $\sqrt{{s}_{NN}}$ = 200 GeV. Switching from central to peripheral collisions: the kinetic freeze-out temperature (*T*_0_) decreases, denotes a change from center to periphery collisions and a longer lifetime of the fireball. Comparison with same data, but different model fitting reveals that *T*_0_ values are increasing from central to peripheral collisions when we use BWTS function, while in case of TS function it gives counter results. Slopes of hadrons’ temperatures in case of TS fittings are negative while positive in case of BWTS function. This trend puts a big question mark on the credibility of using such functions for temperature extraction. After careful analysis we reached to the conclusion that even though there is a discrepancy between different functions concerning temperature, still they can reproduce, for example, *p*_T_ distributions. And since dependency of $\langle \beta_{{\text{T}}} \rangle$ on centrality reveals decreasing behavior in cases of both functions, so we can say that both fitting functions, TS and BWTS may be used to extract temperature of hadrons.

The decrease in *T*_0_ values for hadrons is greater in the TS case than the increase in values of *T*_0_ in the BWTS case. The decrease in slopes of hadrons is particle mass dependent. The greater the mass, greater the slope reduction. Kinetic freeze-out temperature depends on mass of the particles. It increases with increasing particle mass. It also means that heavier particles arrive to the freeze-out phase before their lighter counterparts do. Drop in centrality causes a drop in *N* and *n* values.

## Data Availability

The study's supporting data has been included into the article and is correctly cited in the text at pertinent places.
